# Efficacy and safety of doravirine/lamivudine/tenofovir as initial treatment for people living with HIV in China

**DOI:** 10.1128/aac.00074-26

**Published:** 2026-06-04

**Authors:** Qianhui Chen, Yanhe Luo, Qi Zheng, Miao Tan, Songjie Wu, Shi Zou, Yuting Tan, Jie Liu, Shihui Song, Qian Du, Ke Hong, Ke Liang

**Affiliations:** 1 Department of Infectious Diseases, Zhongnan Hospital of Wuhan University 89674, Wuhan, China; 2 Department of Infectious Diseases, Wuhan Jinyintan Hospital, Tongji Medical College of Huazhong University of Science and Technology 194046, Wuhan, China; 3 Hubei Clinical Research Center for Infectious Diseases 603220 , Wuhan, China; 4 Wuhan Research Center for Communicable Disease Diagnosis and Treatment, Chinese Academy of Medical Sciences 71046, Wuhan, China; 5 Joint Laboratory of Infectious Diseases and Health, Wuhan Institute of Virology and Wuhan Jinyintan Hospital, Chinese Academy of Sciences 74614 , Wuhan, China; 6 Department of Nosocomial Infection Management, Zhongnan Hospital of Wuhan University 89674, Wuhan, China; Chinese Academy of Medical Sciences & Peking Union Medical College 617896, Beijing, China

**Keywords:** doravirine/lamivudine/tenofovir, HIV, initial treatment, antiviral efficacy, lipid profiles

## Abstract

The newly approved oral anti-HIV doravirine (DOR)-based regimen in China in treatment-naïve people living with HIV (PLWH) remains inadequately characterized in China, particularly regarding antiviral efficacy and lipid profile changes. This study aimed to assess 48-week antiviral effectiveness and lipid changes associated with a DOR-based regimen in treatment-naïve PLWH compared with other regimens. A real-world retrospective study was conducted involving treatment-naïve PLWH who initiated DOR/TDF/3TC, EFV/TDF/3TC, DTG/TDF/3TC, or BIC/TAF/FTC. At week 48, 96.22% of participants receiving DOR/TDF/3TC achieved viral suppression, accompanied by significant increases in CD4^+^ T-cell counts. No treatment-related adverse events were reported in the DOR group. There were no statistically significant differences in virologic suppression or CD4^+^ T-cell increases among the four regimens (all *P* > 0.05). Regarding lipid parameters, DOR/TDF/3TC was associated with significant reductions in total cholesterol (TC) and triglycerides (TG) at weeks 36 and 48 (all *P* < 0.05), whereas BIC/TAF/FTC showed significant increases at all four post-treatment timepoints in TG, TC, and low-density lipoprotein cholesterol (LDL-C) (all *P* < 0.05). Between-group comparisons showed lower levels with DOR group versus BIC group in TG, TC, and LDL-C at week 48 (*P* < 0.05). DOR showed lower levels in TC than EFV across the four time points (all *P* < 0.05). DOR/TDF/3TC demonstrated antiviral effectiveness comparable to DTG/TDF/3TC and BIC/TAF/FTC, with a favorable tolerability profile at week 48. Additionally, DOR/TDF/3TC was associated with more favorable lipid changes.

## INTRODUCTION

With the widespread adoption of antiretroviral therapy (ART), the life expectancy of people living with HIV (PLWH) has markedly increased. However, chronic comorbidities, such as cardiovascular disease and metabolic syndrome, have become prominent public health concerns. Dyslipidemia, one of the major long-term adverse effects associated with ART, is a critical risk factor for these conditions (1–4). Evidence indicates that ART is associated with dyslipidemia, and different regimens appear to drive distinct alterations in lipid metabolism. Notably, whether due to genetic factors, ART regimen, or HIV infection itself, the prevalence of dyslipidemia is consistently higher in HIV-infected populations (5). Therefore, elucidating the specific effects of different ART regimens on lipid profiles and selecting appropriate treatments are crucial to optimize long-term health management in PLWH.

The efavirenz (EFV)/tenofovir disoproxil fumarate (TDF)/lamivudine (3TC) regimen was previously considered a global first-line regimen. With the availability of newer and safer agents, limitations, such as central nervous system toxicity, have become increasingly apparent, and current international and domestic guidelines generally classify EFV/TDF/3TC as an alternative or second-line option (6–9). Nevertheless, due to constrained drug availability and cost considerations, EFV/TDF/3TC remains recommended and readily accessible under China’s national free ART program (10). Prior reports indicated that more than 50% of PLWH nationwide, and approximately 70% in Shenzhen, initiated ART with TDF/3TC/EFV (11, 12). In recent years, integrase strand transfer inhibitor (INSTI)-based regimens have become the preferred first-line options owing to superior efficacy and favorable safety profiles, including dolutegravir (DTG)-based combinations with TDF or tenofovir alafenamide (TAF) plus 3TC, and bictegravir (BIC)/TAF/emtricitabine (FTC) (6–9). Although outcomes for PLWH have continued to improve, persistent concerns about tolerability, such as hepatotoxicity, dyslipidemia, and neuropsychiatric symptoms, sustain the need for novel antiretroviral agents across existing and new drug classes.

The advent of novel non-nucleoside reverse transcriptase inhibitors (NNRTIs) has provided new therapeutic options. Doravirine (DOR), a next-generation NNRTI, is indicated for the treatment of HIV infection (13). It is available as a fixed-dose combination tablet with the nucleoside reverse transcriptase inhibitors (NRTIs) 3TC and TDF in the United States, China, Africa, and other regions (6, 14, 15). Clinical trials and meta-analyses have shown that DOR/TDF/3TC is an effective, safe, and well-tolerated option for initial treatment and for virologically suppressed PLWH switching regimens (16). However, real-world evidence from China lacks direct comparisons between DOR/TDF/3TC and the main guideline-recommended regimens used in China. As a result, the relative effectiveness and safety profile of DOR/TDF/3TC remain to be fully established. This real-world, retrospective cohort study aims to compare the 48-week effectiveness and safety of DOR/TDF/3TC versus three commonly used three-drug regimens (EFV/TDF/3TC, DTG/TDF/3TC, and BIC/TAF/FTC) in ART-naïve PLWH in China and further evaluate the impact of DOR/TDF/3TC on lipid profiles, thereby providing evidence to inform optimization of clinical ART strategies.

## MATERIALS AND METHODS

### Study design and participants

This retrospective real-world cohort enrolled treatment-naïve adults with HIV who attended Wuhan Jinyintan Hospital, China and were followed up at the clinic. Eligibility was assessed among patients initiating ART between August 2023 and August 2024. Inclusion criteria were: (i) age ≥18 years; (ii) confirmed HIV infection with no prior exposure to antiretroviral drugs; and (iii) initiation of one of the following regimens: DOR/TDF/3TC (DOR group), EFV/TDF/3TC (EFV group), DTG/TDF/3TC (DTG group), or BIC/TAF/FTC (BIC group). Exclusion criteria included pregnancy or lactation, severe hepatic and renal impairment, significant immunological disorders, psychiatric conditions, and active tuberculosis co-infection. All participants provided written informed consent. This study was approved by the Ethics Committee of Wuhan Jinyintan Hospital (no. KY-2025-12).

### Procedures and data collection

Patients were followed up at 12-week intervals. Data were extracted from the China Information System for Disease Control and Prevention and included demographics (age, sex, height, weight, sexual orientation, body mass index [BMI], marital status, comorbidities, and concomitant medications, including lipid-lowering agents), HIV-related information (date of diagnosis, transmission route, comorbidities, plasma HIV RNA level, and CD4^+^ T-cell count), and laboratory parameters (hematology, liver enzymes, total bilirubin [T.BIL], direct bilirubin [D.BIL], blood glucose, uric acid, serum creatinine, and lipid profiles: total cholesterol [TC], triglycerides [TG], high-density lipoprotein cholesterol [HDL-C], and low-density lipoprotein cholesterol [LDL-C]). The lipid measurements were obtained under fasting conditions. HIV RNA was assessed every 24 weeks, CD4^+^ T-cell counts annually, and all other parameters every 12 weeks. Safety was evaluated by participant self-report and investigator assessment. Adverse events (AEs) were recorded, including clinical symptoms, vital sign abnormalities, and laboratory deviations. Treatment failure was defined as failure to achieve virologic suppression, with HIV RNA >200 copies/mL at week 24. Liver dysfunction was defined as transaminase or bilirubin levels exceeding twice the upper limit of normal.

### Study endpoints

The observation period was 48 weeks. The primary endpoint was the proportion of participants with HIV RNA ≤50 copies/mL at week 48. Secondary endpoints were the change in CD4^+^ T-cell count at week 48 and changes in lipid parameters (TC, TG, HDL-C, LDL-C) at weeks 12, 24, 36, and 48. The safety endpoint was the incidence of AEs over 48 weeks.

### Statistical analysis

Continuous variables were presented as means ± standard deviations (SD) or medians (interquartile range [IQR]) based on distribution normality. Categorical variables were described as counts and percentages, and nonparametric tests (Kruskal-Wallis) were used for statistical analysis. Paired Student’s *t*-tests were used for intragroup comparisons. Intergroup comparisons were performed using the R project (linear mixed-effects model). The Kenward-Roger method was utilized to adjust denominator degrees of freedom for tests of fixed effects. For pairwise comparisons between treatment groups, Tukey’s honestly significant difference (HSD) procedure was applied to adjust *P*-values. All tests were two-tailed with statistical significance defined as *P* < 0.05. Statistical analysis was performed using GraphPad Prism version 9.4 (GraphPad Software, San Diego, CA, USA).

## RESULTS

### Patient characteristics

**Fig 1:**
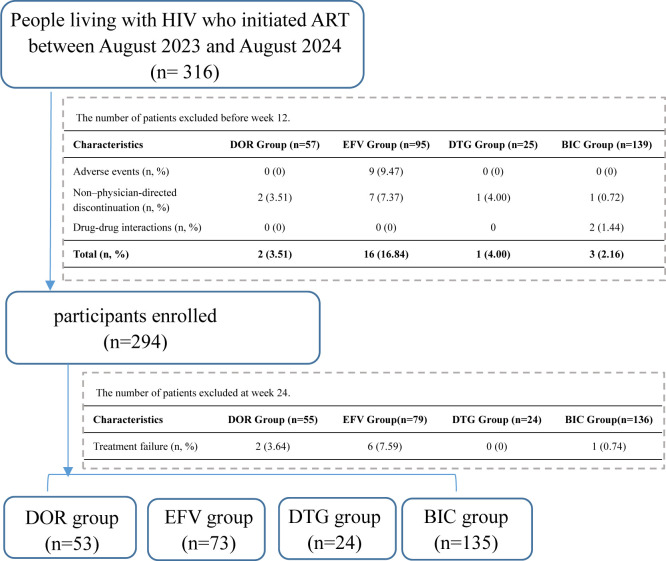
Flowchart of the study participant enrollment.

A total of 316 PLWH initiated ART between August 2023 and August 2024 with one of the following regimens: DOR/TDF/3TC (*n* = 57), EFV/TDF/3TC (*n* = 95), DTG/TDF/3TC (*n* = 25), or BIC/TAF/3TC (*n* = 139). Twenty-two PLWH were excluded due to regimen modification or discontinuation attributable to AEs, drug-drug interactions, and non-physician-directed discontinuation across the treatment groups. Furthermore, the EFV group showed the numerically highest proportion of 24-week treatment failure rate (7.59%) with no statistically significant difference among groups (DOR 3.64%, DTG 0%, BIC 0.74%) at week 24. The final analytic cohort comprised 53 patients in the DOR group, 73 in the EFV group, 24 in the DTG group, and 135 in the BIC group at week 48 (Fig. 1).

No significant differences were observed among the four groups in sex, sexual orientation, marital status, transmission route, or comorbidities. As shown in Table 1, most participants were male. The groups had comparable prevalences of diabetes mellitus, cardiovascular and cerebrovascular diseases, cancer, femoral head necrosis, hepatitis B virus (HBV) infection, hepatitis C virus (HCV) infection, and syphilis. In contrast, age and body mass index (BMI) differed significantly across groups: the EFV group was younger than the BIC (*P* = 0.003) and DOR (*P* = 0.028) groups, and the DOR group had a higher BMI than the EFV (*P* = 0.010) and BIC (*P* = 0.017) groups. Although baseline alanine aminotransferase (ALT) and aspartate aminotransferase (AST) levels differed significantly, the proportion with hepatic impairment did not vary across groups. Baseline immunovirologic measures also varied (Table 1): CD4^+^ T-cell count and plasma HIV RNA levels differed significantly across groups. The DOR group had a higher CD4^+^ T-cell count (vs DTG: *P* = 0.023) and lower HIV RNA levels (vs DTG: *P* = 0.025; vs BIC: *P* = 0.003) (not shown in Table 1).

**TABLE 1:** Baseline demographics and clinical characteristics for patients completing 48 weeks of follow-up^*a*^

Characteristics	DOR group (*n* = 53)	EFV group (*n* = 73)	DTG group (*n* = 24)	BIC group (*n* = 135)	*P* value
Age, years	36 (30–42)	28 (20–49)	41 (28–51)	34 (27–56)	0.002
Male, *n* (%)	49 (92.40%)	68 (93.15%)	20 (83.33%)	125 (92.50%)	0.733
Marital status, *n* (%)					0.525
Single	42 (79.25%)	63 (86.30%)	19 (79.17%)	113 (83.70%)	
Married	11 (20.70%）	10 (13.70%)	5 (20.83%)	22 (16.20%)	
Sexual orientation, *n* (%)					0.067
Homosexuality	28 (52.80%)	50 (68.49%)	11 (45.83%)	80 (59.20%)	
Heterosexuality	25 (47.10%)	23 (31.51%)	13 (54.17%)	55 (40.70%)	
Transmission route, *n* (%)					0.886
Sexual	53 (100%)	72 (98.63%)	23 (95.83%)	134 (99.20%)	
Sexual and drug use	0 (0%)	1 (1.37%)	1 (4.17%)	1 (0.70%)	
BMI (kg/m²^2^)	23.84 (21.31–25.51)	21.63 (19.61–23.95)	23.07 (20.95–25.60)	22.13 (19.35–24.49)	0.005
Comorbidity, *n* (%)	5	0	4	11	0.444
Diabetes mellitus	5 (9.40%)	0	2 (8.33%)	7 (5.10%)	
Cardiovascular and cerebrovascular diseases	0 (0%)	0	1 (4.17%)	2 (1.40%)	
Tumors	0 (0%)	0	1 (4.17%)	2 (1.40%)	
Femoral head necrosis	0 (0%)	0	0 (0%)	0 (0%)	
HBV infection	2 (3.70%)	3 (4.11%)	1 (4.17%)	10 (7.40%)	
HCV infection	0 (0%)	1 (1.37%)	0 (0%)	5 (3.70%)	
Syphilis	15 (28.30%)	26 (35.62%)	7 (29.17%)	31 (22.90%)	
Diagnosis-to-treatment interval (days)	18 (12–25)	15 (12–27)	13 (10.25–20.50)	16 (12–26.50)	0.316
CD4 count (cells/μL)	283 (179–380)	245 (161–346)	184.50 (52.75–291.75)	240 (92–367)	0.030
HIV RNA (Log₁₀ copies/mL)	4.52 (4.05–4.93)	4.83 (4.31–5.22)	5.15 (4.38–5.58)	5.01 (4.30–5.40)	0.002
White blood cells (10^9/L)	5.61 ± 1.45	5.40 ± 1.36	5.20 ± 1.63	5.29 ± 1.86	0.639
Platelets (10^9/L)	226.30 ± 59.55	222.52 ± 61.76	225.54 ± 91.09	204.91 ± 69.56	0.119
Hemoglobin (g/L)	145.83 ± 14.89	143.55 ± 16.86	132.42 ± 16.20	139.41 ± 20.08	0.010
Serum creatinine (μmol/L)	73.17 ± 11.64	72.85 ± 10.83	67.80 ± 12.83	71.97 ± 12.14	0.270
Serum uric acid (μmol/L)	425.58 ± 99.38	426.90 ± 120.40	408.32 ± 110.20	402.71 ± 105.93	0.404
Fasting blood glucose (mmol/L)	6.21 ± 3.02	5.72 ± 1.27	6.44 ± 2.61	5.90 ± 1.86	0.394
ALT (U/L)	42.75 ± 60.05	22.88 ± 14.02	33.67 ± 33.64	33.07 ± 29.81	0.005
AST (U/L)	31.81 ± 33.48	22.96 ± 7.44	31.88 ± 23.66	29.52 ± 26.17	0.033
T.BIL (μmol/L)	16.89 ± 27.78	12.51 ± 4.79	11.98 ± 4.46	11.85 ± 5.01	0.436
D.BIL (μmol/L)	7.51 ± 26.09	3.98 ± 1.75	3.76 ± 1.09	3.74 ± 1.88	0.697
Liver dysfunction (*n*, %)	10 (18.87%)	4 (5.48%)	3 (12.50%)	17 (12.59%)	0.771

^*a*^ Data are presented as median, interquartile range, or *n* (%). BMI, body mass index; CD4 count, CD4^+^ T lymphocyte count; ALT, alanine aminotransferase; AST, aspartate aminotransferase; T.BIL, total bilirubin; D.BIL, direct bilirubin.

### Changes in HIV RNA levels and CD4 count at week 48 after ART

HIV RNA levels were assessed as the primary endpoint after treatment initiation. All four groups achieved significant virologic suppression from baseline to week 48 (*P* < 0.001). However, the proportion of PLWH achieving HIV RNA <50 copies/mL at week 48 did not differ significantly across groups (DOR, 96.22%; EFV, 98.63%; DTG, 100%; BIC, 95.56%; *P* = 0.7714; Table 2).

**TABLE 2:** Virologic outcomes and CD4 count changes at week 48

Characteristics	DOR group	EFV group	DTG group	BIC group	*P* value
Plasma HIV RNA (copies/mL)					
≥100,000	0 (0%)	0 (0%)	0 (0%)	0 (0%)	
200–99,000	0 (0%)	0 (0%)	0 (0%)	4 (2.96%)	
50–199	2 (3.80%)	1 (1.37%)	0 (0%)	2 (1.48%)	
＜50	51 (96.22%)	72 (98.63%)	24 (100%)	129 (95.56%)	0.771
Change of CD4 count from baseline (cells/μL)	189 (51–254)	192 (90.75–290)	129.50 (65.50–238)	158 (78–262)	0.052
>30% increase from baseline (*n*, %)	36 (67.90%)	55 (75.34%)	18 (75%)	103 (76.30%)	0.410
>100 cells/µL increase from baseline (*n*, %)	34 (64.20%)	49 (67.12%)	16 (66.67%)	89 (65.92%)	0.114

All four groups showed significant increases in CD4^+^ T-cell count from baseline to week 48 (*P* < 0.001). However, the median change in CD4^+^ T-cell count from baseline to the endpoint did not differ significantly across groups. Likewise, there were no significant between-group differences in the proportion with a ≥30% increase (DOR 67.9%; EFV 75.34%; DTG 75%; BIC 76.30%; *P* = 0.410; Table 2) or an absolute increase of ≥100 cells/µL (DOR 64.2%; EFV 67.12%; DTG 66.67%; BIC 65.92%; *P* = 0.114; Table 2) in CD4^+^ T-cell count from baseline.

As shown in Table 3, no significant changes from baseline were observed at the 48-week follow-up in ALT, AST, total bilirubin (T.BIL), direct bilirubin (D.BIL), fasting blood glucose, serum creatinine, white blood cell count, hemoglobin, or platelet count (all *P* > 0.05), whereas serum uric acid decreased significantly (*P* = 0.010). No moderate or severe AEs were reported. Overall, the safety analysis of the DOR/TDF/3TC regimen indicated favorable tolerability.

**TABLE 3:** Comparisons of safety indicators for DOR/3TC/TDF at week 48 versus baseline

Characteristics	Baseline	Week 48	*P* value
ALT (U/L)	26 (19–38)	27 (20–48)	0.866
AST (U/L)	23 (19–27)	27 (20–33)	0.916
T.BIL (μmol/L)	12.90 (9.40–15.60)	11.5 (8.50–16.30)	0.311
D.BIL (μmol/L)	3.60 (2.60–4.60)	3.40 (2.50–4.20)	0.278
Blood glucose (mmol/L)	5.58 (4.99–5.26)	5.80 (5.33–6.31)	0.946
Serum creatinine (μmol/L)	72.20 (67.10–82.50)	78.3 (69.10–84.40)	0.145
Serum uric acid (μmol/L)	430 (360–499)	382 (323–430)	0.010
White blood cells (10^9^/L)	5.49 (4.60–6.20)	6.12 (5.45–7.13)	0.033
Platelets (10^9^/L)	221 (186–258)	229 (198–272)	0.456
Hemoglobin (g/L)	146 (137–158)	154 (145–160)	0.009

### BMI changes after ART

**Fig 2:**
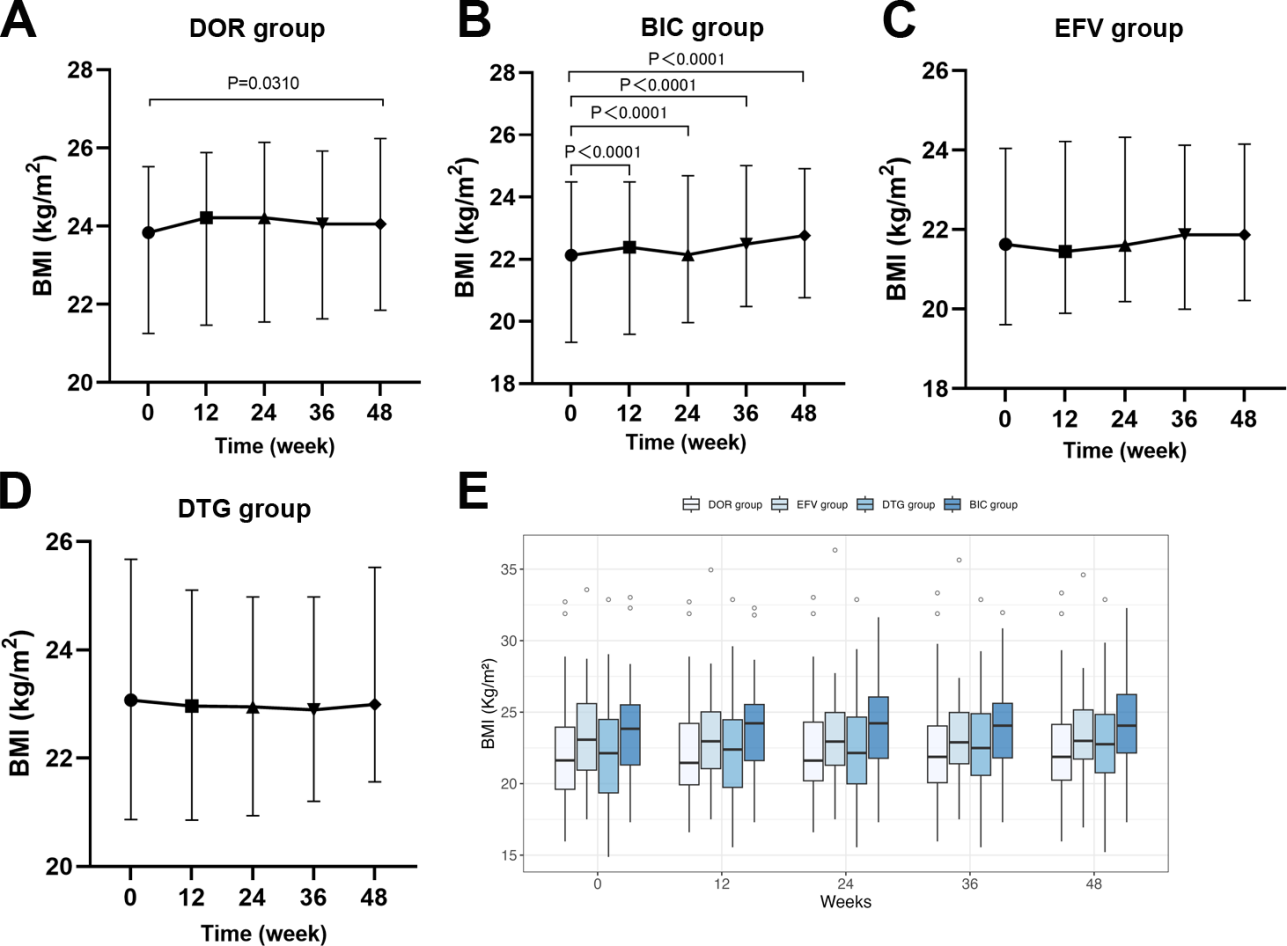
BMI changes after ART at different time points. BMI changes at week 12, 24, 36, and 48 for the DOR (**A**), BIC (**B**), EFV (**C**), and DTG groups (**D**). Paired Student’s *t*-tests were used for intragroup comparisons. Data distribution for BMI across the four groups throughout the 48-week study (**E**). Intergroup comparisons were performed using the linear mixed-effects model.

We first compared BMI changes over time within each treatment group. In the DOR group, a significant BMI increase was observed at week 48 relative to baseline (*P* = 0.013) with no significant differences at earlier time points (all *P* > 0.05) (Fig. 2A). In the BIC group, BMI increased significantly at weeks 12, 24, 36, and 48 compared with baseline (all *P* < 0.001) (Fig. 2B). By contrast, in the EFV and DTG groups, no significant differences from baseline were observed at any post-treatment time points (weeks 12, 24, 36, 48) (all *P* > 0.05) (Fig. 2C and D).

Intergroup differences in BMI were evaluated at 12-week intervals up to week 48 among the four groups using linear mixed models with baseline adjustment. Data distribution for BMI across the four groups throughout the 48-week study was shown in Fig. 2E and Table S1. There were no significant between-group differences at the 12- or 24-week post-treatment assessments (all *P* > 0.05). By contrast, the BMI levels were significantly higher in the BIC group compared with the EFV and DTG groups at weeks 36 (*P* = 0.022 and *P* = 0.002, respectively) and 48 (*P* = 0.001 and *P* = 0.005, respectively), whereas the remaining intergroup comparisons showed no significant differences (all *P* > 0.05).

### Within-group changes in lipid levels

**Fig 3:**
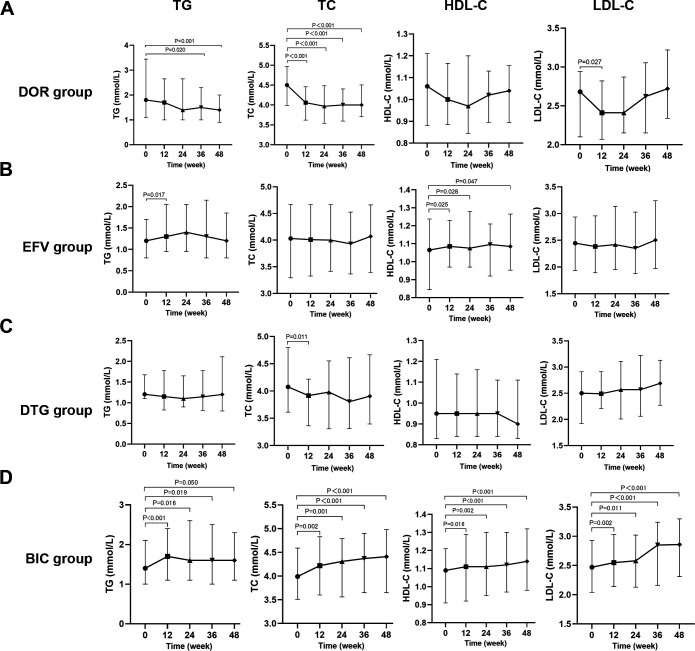
Within-group changes in lipid levels at different time points. Changes in TG, TC, HDL-C, and LDL-C at weeks 12, 24, 36, and 48 for the DOR (**A**), EFV (**B**), DTG (**C**), and BIC groups (**D**). TC, total cholesterol; TG, triglycerides; HDL-C, high-density lipoprotein cholesterol; and LDL-C, low-density lipoprotein cholesterol. Paired Student’s *t*-tests were used for intragroup comparisons.

In the DOR group (Fig. 3A), TG decreased significantly at weeks 36 and 48 relative to baseline (*P* = 0.020 and *P* < 0.001, respectively) with no significant differences at week 12 or 24 (all *P* > 0.05). TC decreased significantly at all post-treatment time points (weeks 12, 24, 36, and 48) compared with baseline (all *P* < 0.001). LD-C decreased significantly only at week 12 (*P* = 0.027) with no significant differences at week 24, 36, or 48 (all *P* > 0.05). HDL-C showed no significant changes from baseline at any post-treatment time point (weeks 12, 24, 36, and 48) (all *P* > 0.05).

In the EFV group (Fig. 3B), TG increased significantly only at week 12 relative to baseline (*P* = 0.017) with no significant differences at week 24, 36, or 48 (all *P* > 0.05). HDL-C increased significantly at weeks 12, 24, and 48 (*P* = 0.025, *P* = 0.028, and *P* = 0.047, respectively) with no significant difference at week 36 (*P* > 0.05). By contrast, TC and LDL-C showed no significant changes from baseline at any post-treatment time point (weeks 12, 24, 36, and 48) (all *P* > 0.05).

In the DTG group (Fig. 3C), TC decreased significantly only at week 12 compared with baseline (*P* = 0.011) with no significant differences at week 24, 36, or 48 (*P* > 0.05). TG, HDL-C, and LDL-C showed no significant changes from baseline at any post-treatment time point (weeks 12, 24, 36, and 48) (all *P* > 0.05).

In the BIC group (Fig. 3D), significant increases from baseline were observed at all post-treatment time points (weeks 12, 24, 36, and 48) in TG (*P* < 0.001, *P* = 0.016, *P* = 0.019, and *P* = 0.050, respectively), TC (*P* = 0.002, *P* = 0.001, *P* < 0.001, and *P* < 0.001, respectively), HDL-C (*P* = 0.016, *P* = 0.002, *P* < 0.001, and *P* < 0.001, respectively), and LDL-C (*P* = 0.002, *P* = 0.011, *P* < 0.001, and *P* < 0.001, respectively).

### Between-group comparisons of lipid levels at different time points

**Fig 4:**
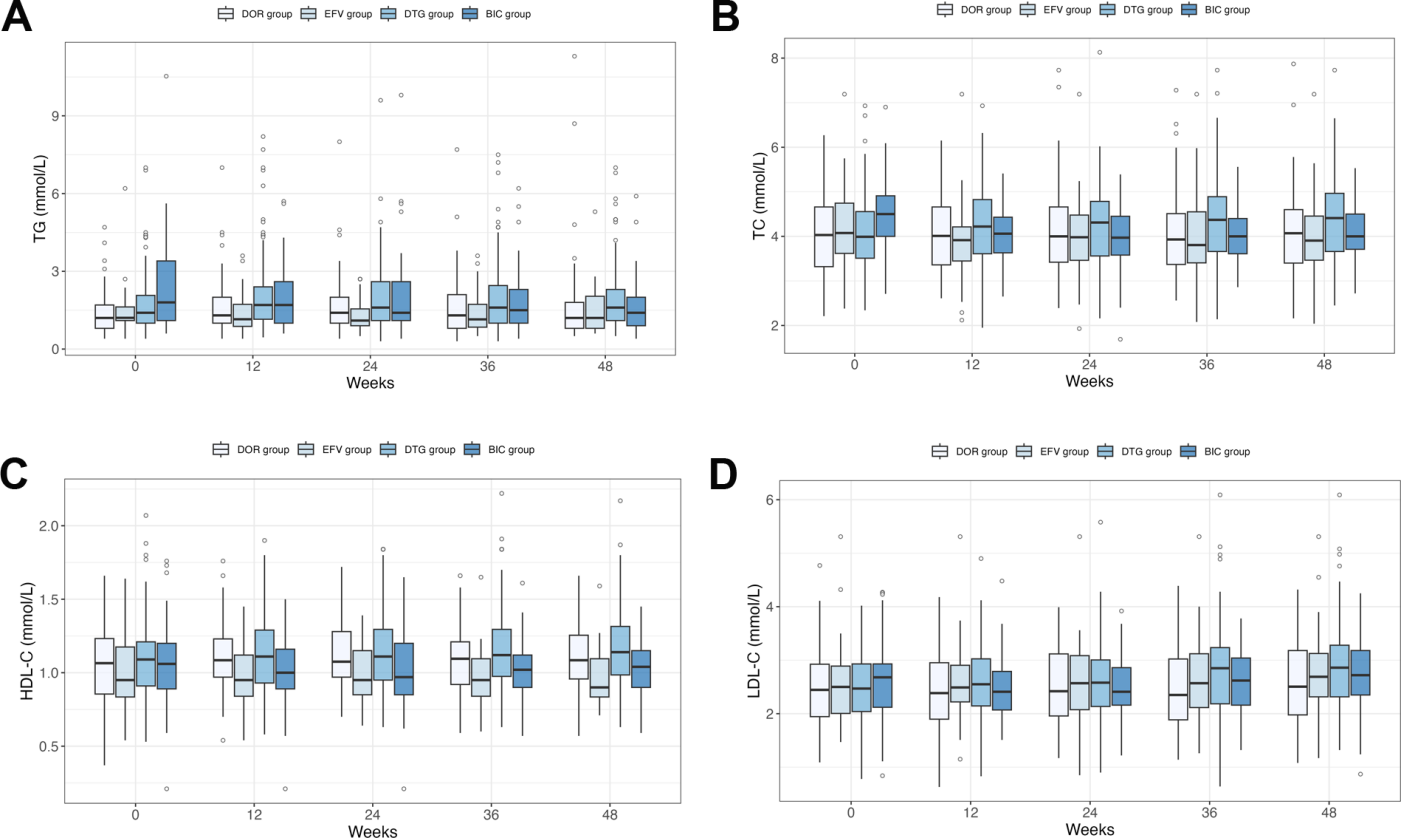
Data distribution for lipid levels across the four groups throughout the 48-week study. Data distribution for TG (**A**), TC (**B**), HDL-C (**C**), and LDL-C (**D**) at 12-week intervals through 48 weeks across all four groups. TC, total cholesterol; TG, triglycerides; HDL-C, high-density lipoprotein cholesterol; and LDL-C, low-density lipoprotein cholesterol. Intergroup comparisons were performed using the linear mixed-effects model.

We compared the lipid levels at weeks 12, 24, 36, and 48 across the four groups after baseline adjustment. Data distribution for TG, TC, HDL-C, and LDL-C across the four groups throughout the 48-week study is shown in Fig. 4 and Table S1. Significantly higher TG levels were observed in the BIC group compared with the DOR group at week 48 (*P* = 0.010). None of the remaining between-group comparisons for TG levels at other time points were significant (all *P* > 0.05).

Significantly lower TC levels were observed in the DOR group compared with the EFV group (*P* = 0.020, *P* < 0.001, *P* = 0.018, and *P* = 0.003, respectively) and BIC group (all *P* < 0.001) at all post-treatment time points (weeks 12, 24, 36, and 48). Additionally, significantly lower TC levels were observed in the DTG group compared with the BIC group at weeks 12, 36, and 48 (*P* = 0.048, *P* = 0.004, and *P* = 0.007, respectively) with no significant difference at week 24 (*P* > 0.05). None of the remaining between-group comparisons for TC levels at other time points were significant (all *P* > 0.05).

Significantly higher HDL-C levels were observed in the EFV group compared with the DTG group at weeks 12, 24, and 48 (*P* = 0.020, *P* = 0.019, and *P* = 0.008, respectively). In addition, HDL-C levels in the BIC group were significantly higher than those in the DTG (*P* = 0.021, *P* = 0.018, *P* = 0.003, and *P* < 0.001, respectively) and DOR groups (*P* = 0.043, *P* = 0.013, *P* = 0.009, and *P* = 0.008, respectively) at all post-treatment time points (weeks 12, 24, 36, and 48). Significantly higher HDL-C levels were only observed in the EFV group compared with the DOR group at weeks 12 and 24 (*P* = 0.045 and *P* = 0.017). None of the remaining between-group comparisons for HDL-C levels at other time points were significant (all *P* > 0.05).

LDL-C levels in the BIC group were significantly higher than those in the EFV (*P* < 0.001 and *P* = 0.014, respectively) and DOR groups (*P* = 0.012 and *P* = 0.038, respectively) at weeks 36 and 48. None of the remaining between-group comparisons for LDL-C levels at other time points were significant (all *P* > 0.05).

## DISCUSSION

In this real-world, retrospective cohort study, the DOR group showed potent HIV RNA suppression and significant CD4^+^ T-cell count increases, along with notable reductions in lipid parameters (TG, TC, and LDL-C) at week 48. Furthermore, its antiviral efficacy and CD4 recovery were comparable to those achieved with the DTG and BIC groups. In terms of safety, the DOR/TDF/3TC regimen demonstrated favorable tolerability.

The approval of DOR for treatment-naïve patients was supported primarily by two Key Phase 3 trials: DRIVE-AHEAD and DRIVE-FORWARD (17, 18). In DRIVE-AHEAD, among 728 treatment-naïve patients, virologic suppression at week 48 was 80.8% with EFV/FTC/TDF and 84.3% with DOR/3TC/TDF (17). Similarly, DRIVE-FORWARD showed a 48-week suppression rate of 84% for DOR with two NRTIs versus 80% for darunavir/ritonavir (18). The Phase 3 DRIVE-SHIFT trial further demonstrated maintained suppression in patients who switched from a stable regimen to DOR/3TC/TDF (19). Consistent with these data, our study found that DOR-based therapy achieved virologic suppression and CD4^+^ T-cell recovery comparable to DTG- and BIC-based regimens. We also confirmed reliable antiviral efficacy for EFV-based therapy among participants who did not switch treatments at week 48. Notably, this analysis excluded nine patients with AEs, all involving central nervous system symptoms, seven patients with non-physician-directed discontinuation, and six with treatment failure. The EFV group had the numerically highest number and proportion of switches due to AEs, non-physician-directed discontinuation, and treatment failure, leading to their exclusion, which aligns with evidence that the declining use of EFV-based regimens as first-line therapy is driven by AEs, particularly long-term neuropsychiatric toxicity, and increased resistance risk (6–9, 20, 21).

Although resistance testing was not performed in our cohort, prior evidence indicates that DOR/TDF/3TC remains an attractive NNRTI-based option with a higher genetic barrier to resistance for initial therapy (22–24). Global analyses show that more than 80% of transmitted drug resistance (TDR) sequences harbor a single NNRTI drug-resistance mutation (DRM), and over 90% with a single DRM are predicted to remain susceptible to DOR (22). These data suggest that a first-line regimen containing DOR plus two NRTIs is likely to be effective in most patients with TDR (22). In a study from Poland, the detection rate of DOR resistance was 1.62%, including 0.74% in naïve and 9.50% in treatment-experienced patients (23). Among naïve patients, resistance to DOR (0.7%) was less common than to nevirapine (NVP) (2.1%) or rilpivirine (RPV) (5.4%) (23). Similarly, a study from China reported a lower prevalence of resistance to DOR (1.8%) than to NVP (5.2%), RPV (3.5%), EFV (3.4%), or etravirine (2.7%) among treatment-naïve individuals in Hebei province (24). Collectively, this evidence supports DOR/TDF/3TC as a highly effective option for most treatment-naïve patients, including in China, thereby reinforcing the role of DOR in first-line therapy. Although the external data support the potential utility of DOR/TDF/3TC in regions with limited access to baseline resistance testing, it is important to emphasize that the baseline resistance testing was not performed in our study. Based on our findings, we cannot draw resistance-related conclusions in ART-naïve PLWH in China. Nonetheless, baseline resistance testing remains advisable before initiating therapy to identify the rare individuals at high risk due to complex multidrug-resistant mutations and maximize the clinical value of DOR/TDF/3TC.

Furthermore, one of the most interesting findings of this study was the favorable impact of the DOR-based regimen on the lipid profile. Our study revealed that the DOR group showed significant lower levels in key lipid parameters, including TG and TC. Notably, compared with the BIC-based regimen, which was associated with increases in these parameters from baseline, the lipid-lowering effect of DOR was both statistically significant and clinically meaningful. This distinction carries substantial implications for long-term patient management. As the life expectancy of PLWH approaches that of the general population, non-AIDS-defining comorbidities, particularly cardiovascular disease (CVD), have become a leading cause of morbidity and mortality (25). Dyslipidemia is a well-established, major modifiable risk factor for CVD (26). Therefore, an ART regimen that combines excellent virologic efficacy with improvement in lipid abnormalities represents a meaningful therapeutic advantage. The DOR regimen, as evidenced by our data, appears to fulfill this dual objective. Research has indicated that the co-formulation of BIC and TAF has a neutral to negative impact on lipid profiles, and TAF itself is known to cause mild lipid elevations compared with its predecessor, TDF (27–30). Our findings confirm this trend. In contrast, DOR, as a novel NNRTI, has demonstrated a metabolically benign or even beneficial profile in clinical trials (13, 17), which is strongly supported by our real-world data. This differential effect critically informs personalized treatment strategies. For PLWH with dyslipidemia or a family history of CVD, initiating or switching to a DOR-based regimen represents a proactive approach to optimizing both virologic and cardiovascular outcomes. It may reduce the need for lipid-lowering drugs, thereby simplifying therapy and helping to avoid polypharmacy.

In addition, a recent Phase 3 trial analyzed factors associated with weight changes in individuals who continued or switched to DOR-based regimen (31). The findings indicated that switching to DOR was generally weight neutral, with the majority of participants (>57%) maintaining stable weight (<5% change). However, the study also noted that weight changes may vary depending on demographic characteristics and the weight-suppressive effects of prior regimens. In our study, DOR/TDF/3TC regimen did not lead to significant weight gain at week 36. Nevertheless, a modest but statistically significant increase in BMI from baseline to week 48 was observed. Several factors may account for this discrepancy. In our treatment-naïve population, the observed increase in BMI likely reflects, at least in part, a return-to-health phenomenon. Nevertheless, the potential confounding effect of the small sample size cannot be ruled out, and larger-scale studies are needed to validate this finding. Notably, the DTG/TDF/3TC regimen demonstrated the lowest rate of treatment failure at week 24. Compared with the BIC group, the three TDF-containing regimens (the EFV, DTG, and DOR groups) all appeared to exhibit favorable metabolic effects, with relatively mild impacts on body weight and lipid parameters. It may, therefore, be inferred that these findings could be attributed to the effects of TDF in reducing lipid levels and body weight. However, some studies have reported that this phenomenon may instead represent a toxic side effect resulting from TDF-induced damage to duodenal villi (32). In addition, TDF has also been associated with nephrotoxicity and an increased risk of bone mineral density loss (33, 34). Thus, regimen selection requires careful consideration of individual patient characteristics, virologic efficacy, metabolic safety, and long-term organ toxicity.

Several limitations of our study should be noted, including inherent risks of bias associated with its retrospective design, single-center data collection, inclusion of only treatment-naïve participants without prior regimen changes, and a relatively small sample size. Nevertheless, our findings are supported by multiple global prospective clinical trials and real-world studies, which strengthens confidence in the conclusions. Future research with larger sample sizes and longer follow-up periods is needed to further clarify the effects of DOR-based ART regimens on cardiovascular and metabolic outcomes, as well as long-term renal and bone safety, compared with other first-line treatment options. An additional limitation is the absence of drug resistance data. Future studies analyzing DRMs are warranted to provide further evidence for the clinical application of DOR-based ART regimens.

## CONCLUSION

In conclusion, this real-world study supported by the latest evidence demonstrated that DOR-based therapy is effective, safe, and well tolerated in China. The efficacy of DOR/TDF/3TC was non-inferior to DTG/TDF/3TC and BIC/TAF/FTC, with a favorable tolerability profile. In addition, treatment with DOR/TDF/3TC is associated with more favorable lipid changes. Thus, DOR-based therapy represents a highly attractive option for long-term, individualized management of HIV infection, particularly for PLWH with cardiovascular risk factors or metabolic abnormalities.

## Data Availability

Data cannot be shared publicly due to ethical restrictions. Data analyzed in the manuscript will be made available by all the authors.

## SUPPLEMENTAL MATERIAL

Table S1 aac.00074-26-s0001.doc Pairwise comparisons of group differences.

ASM does not own the copyrights to Supplemental Material that may be linked to, or accessed through, an article. The authors have granted ASM a non-exclusive, world-wide license to publish the Supplemental Material files. Please contact the corresponding author directly for reuse.
