# Supplementary material for: Efficacy and safety of doravirine/lamivudine/tenofovir as initial treatment for people living with HIV in China
Source: Antimicrob Agents Chemother. 2026 Jun 4;70(7):e00074-26. doi: 10.1128/aac.00074-26 (PMC13321822; doi:10.1128/aac.00074-26)
Supplement: Table S1 — Pairwise comparisons of group differences. [file aac.00074-26-s0001.doc]

Supplementary Table 1. Pairwise comparisons of group differences.

| Characteristic | Timepoint | Contrast | Estimate | Std. Error | p.value |
| --- | --- | --- | --- | --- | --- |
| BMI | Week 12 | EFV-DTG | 0.1567 | 0.227 | 0.9010 |
|  |  | EFV-BIC | -0.1169 | 0.142 | 0.8426 |
|  |  | EFV-DOR | -0.0379 | 0.176 | 0.9965 |
|  |  | DTG-BIC | -0.2736 | 0.213 | 0.5749 |
|  |  | DTG-DOR | -0.1946 | 0.238 | 0.8463 |
|  |  | BIC-DOR | 0.0790 | 0.158 | 0.9592 |
|  | Week 24 | EFV-DTG | 0.1712 | 0.227 | 0.8749 |
|  |  | EFV-BIC | -0.2162 | 0.142 | 0.4228 |
|  |  | EFV-DOR | -0.0888 | 0.176 | 0.9580 |
|  |  | DTG-BIC | -0.3874 | 0.213 | 0.2673 |
|  |  | DTG-DOR | -0.2600 | 0.238 | 0.6945 |
|  |  | BIC-DOR | 0.1274 | 0.158 | 0.8518 |
|  | Week 36 | EFV-DTG | 0.367 | 0.227 | 0.3715 |
|  |  | EFV-BIC | -0.408 | 0.142 | **0.0217** |
|  |  | EFV-DOR | -0.187 | 0.176 | 0.7114 |
|  |  | DTG-BIC | -0.775 | 0.213 | **0.0018** |
|  |  | DTG-DOR | -0.554 | 0.238 | 0.0935 |
|  |  | BIC-DOR | 0.221 | 0.158 | 0.5030 |
|  | Week 48 | EFV-DTG | 0.178 | 0.227 | 0.8611 |
|  |  | EFV-BIC | -0.531 | 0.142 | **0.0011** |
|  |  | EFV-DOR | -0.380 | 0.176 | 0.1369 |
|  |  | DTG-BIC | -0.710 | 0.213 | **0.0053** |
|  |  | DTG-DOR | -0.558 | 0.238 | 0.0898 |
|  |  | BIC-DOR | 0.152 | 0.158 | 0.7731 |
| TG | Week 12 | EFV-DTG | 0.4394 | 0.257 | 0.3210 |
|  |  | EFV-BIC | -0.0961 | 0.161 | 0.9325 |
|  |  | EFV-DOR | 0.2160 | 0.202 | 0.7069 |
|  |  | DTG-BIC | -0.5356 | 0.241 | 0.1180 |
|  |  | DTG-DOR | -0.2234 | 0.272 | 0.8446 |
|  |  | BIC-DOR | 0.3121 | 0.180 | 0.3078 |
|  | Week 24 | EFV-DTG | 0.5138 | 0.257 | 0.1907 |
|  |  | EFV-BIC | -0.0432 | 0.161 | 0.9932 |
|  |  | EFV-DOR | 0.2108 | 0.202 | 0.7224 |
|  |  | DTG-BIC | -0.5570 | 0.241 | 0.0962 |
|  |  | DTG-DOR | -0.3030 | 0.272 | 0.6814 |
|  |  | BIC-DOR | 0.2540 | 0.180 | 0.4938 |
|  | Week 36 | EFV-DTG | 0.387 | 0.257 | 0.4359 |
|  |  | EFV-BIC | -0.047 | 0.161 | 0.9913 |
|  |  | EFV-DOR | 0.365 | 0.202 | 0.2686 |
|  |  | DTG-BIC | -0.434 | 0.241 | 0.2731 |
|  |  | DTG-DOR | -0.022 | 0.272 | 0.9998 |
|  |  | BIC-DOR | 0.412 | 0.180 | 0.1021 |
|  | Week 48 | EFV-DTG | 0.2486 | 0.257 | 0.7690 |
|  |  | EFV-BIC | -0.0563 | 0.161 | 0.9852 |
|  |  | EFV-DOR | 0.5076 | 0.202 | 0.0580 |
|  |  | DTG-BIC | -0.3049 | 0.241 | 0.5849 |
|  |  | DTG-DOR | 0.2590 | 0.272 | 0.7769 |
|  |  | BIC-DOR | 0.5639 | 0.180 | **0.0099** |
| TC | Week 12 | EFV-DTG | 0.3113 | 0.1550 | 0.1858 |
|  |  | EFV-BIC | -0.0649 | 0.0966 | 0.9077 |
|  |  | EFV-DOR | 0.3484 | 0.1200 | **0.0204** |
|  |  | DTG-BIC | -0.3762 | 0.1450 | **0.0477** |
|  |  | DTG-DOR | 0.0371 | 0.1630 | 0.9958 |
|  |  | BIC-DOR | 0.4133 | 0.1080 | **0.0008** |
|  | Week 24 | EFV-DTG | 0.34732 | 0.1550 | 0.1134 |
|  |  | EFV-BIC | -0.00142 | 0.0966 | 1.0000 |
|  |  | EFV-DOR | 0.49828 | 0.1200 | **0.0002** |
|  |  | DTG-BIC | -0.34875 | 0.1450 | 0.0773 |
|  |  | DTG-DOR | 0.15096 | 0.1630 | 0.7898 |
|  |  | BIC-DOR | 0.49970 | 0.1080 | **<0.0001** |
|  | Week 36 | EFV-DTG | 0.2758 | 0.1550 | 0.2839 |
|  |  | EFV-BIC | -0.2129 | 0.0966 | 0.1235 |
|  |  | EFV-DOR | 0.3534 | 0.1200 | **0.0180** |
|  |  | DTG-BIC | -0.4888 | 0.1450 | **0.0044** |
|  |  | DTG-DOR | 0.0776 | 0.1630 | 0.9642 |
|  |  | BIC-DOR | 0.5663 | 0.1080 | **<0.0001** |
|  | Week 48 | EFV-DTG | 0.292 | 0.1550 | 0.2353 |
|  |  | EFV-BIC | -0.177 | 0.0966 | 0.2582 |
|  |  | EFV-DOR | 0.415 | 0.1200 | **0.0034** |
|  |  | DTG-BIC | -0.469 | 0.1450 | **0.0070** |
|  |  | DTG-DOR | 0.123 | 0.1630 | 0.8747 |
|  |  | BIC-DOR | 0.592 | 0.1080 | **<0.0001** |
| HDL-C | Week 12 | EFV-DTG | 0.12910 | 0.0445 | **0.0199** |
|  |  | EFV-BIC | 0.00902 | 0.0275 | 0.9877 |
|  |  | EFV-DOR | 0.08905 | 0.0340 | **0.0446** |
|  |  | DTG-BIC | -0.12008 | 0.0416 | **0.0208** |
|  |  | DTG-DOR | -0.04004 | 0.0465 | 0.8252 |
|  |  | BIC-DOR | 0.08003 | 0.0304 | **0.0431** |
|  | Week 24 | EFV-DTG | 0.1302 | 0.0445 | **0.0185** |
|  |  | EFV-BIC | 0.0080 | 0.0275 | 0.9914 |
|  |  | EFV-DOR | 0.1008 | 0.0340 | **0.0166** |
|  |  | DTG-BIC | -0.1222 | 0.0416 | **0.0178** |
|  |  | DTG-DOR | -0.0295 | 0.0465 | 0.9214 |
|  |  | BIC-DOR | 0.0928 | 0.0304 | **0.0127** |
|  | Week 36 | EFV-DTG | 0.1075 | 0.0445 | 0.0748 |
|  |  | EFV-BIC | -0.0366 | 0.0275 | 0.5414 |
|  |  | EFV-DOR | 0.0591 | 0.0340 | 0.3049 |
|  |  | DTG-BIC | -0.1441 | 0.0416 | **0.0031** |
|  |  | DTG-DOR | -0.0484 | 0.0465 | 0.7259 |
|  |  | BIC-DOR | 0.0957 | 0.0304 | **0.0093** |
|  | Week 48 | EFV-DTG | 0.1421 | 0.0445 | **0.0079** |
|  |  | EFV-BIC | -0.0271 | 0.0275 | 0.7562 |
|  |  | EFV-DOR | 0.0702 | 0.0340 | 0.1660 |
|  |  | DTG-BIC | -0.1693 | 0.0416 | **0.0003** |
|  |  | DTG-DOR | -0.0720 | 0.0465 | 0.4107 |
|  |  | BIC-DOR | 0.0973 | 0.0304 | **0.0079** |
| LDL-C | Week 12 | EFV-DTG | -0.0868 | 0.1330 | 0.9149 |
|  |  | EFV-BIC | -0.1516 | 0.0820 | 0.2517 |
|  |  | EFV-DOR | 0.0728 | 0.1020 | 0.8906 |
|  |  | DTG-BIC | -0.0648 | 0.1250 | 0.9543 |
|  |  | DTG-DOR | 0.1596 | 0.1390 | 0.6611 |
|  |  | BIC-DOR | 0.2244 | 0.0907 | 0.0651 |
|  | Week 24 | EFV-DTG | -0.0576 | 0.1330 | 0.9728 |
|  |  | EFV-BIC | -0.0893 | 0.0820 | 0.6966 |
|  |  | EFV-DOR | 0.1041 | 0.1020 | 0.7358 |
|  |  | DTG-BIC | -0.0317 | 0.1250 | 0.9942 |
|  |  | DTG-DOR | 0.1617 | 0.1390 | 0.6519 |
|  |  | BIC-DOR | 0.1933 | 0.0907 | 0.1445 |
|  | Week 36 | EFV-DTG | -0.125 | 0.1330 | 0.7838 |
|  |  | EFV-BIC | -0.312 | 0.0820 | **0.0009** |
|  |  | EFV-DOR | -0.034 | 0.1020 | 0.9871 |
|  |  | DTG-BIC | -0.187 | 0.1250 | 0.4359 |
|  |  | DTG-DOR | 0.091 | 0.1390 | 0.9144 |
|  |  | BIC-DOR | 0.278 | 0.0907 | **0.0121** |
|  | Week 48 | EFV-DTG | -0.09328 | 0.1330 | 0.8968 |
|  |  | EFV-BIC | -0.24791 | 0.0820 | **0.0139** |
|  |  | EFV-DOR | -0.00509 | 0.1020 | 1.0000 |
|  |  | DTG-BIC | -0.15463 | 0.1250 | 0.6009 |
|  |  | DTG-DOR | 0.08819 | 0.1390 | 0.9213 |
|  |  | BIC-DOR | 0.24282 | 0.0907 | **0.0382** |
